# Emotional disorder and absence from school: findings from the 2004 British Child and Adolescent Mental Health Survey

**DOI:** 10.1007/s00787-019-01342-4

**Published:** 2019-05-03

**Authors:** Katie Finning, Tamsin Ford, Darren A. Moore, Obioha C. Ukoumunne

**Affiliations:** 1 University of Exeter School of Medicine and Health, College House, St Luke’s Campus, Exeter, EX1 2LU UK; 2 Graduate School of Education, University of Exeter, St Luke’s Campus, Heavitree Road, Exeter, EX1 2LU UK; 3 School of Medicine and Health, University of Exeter, South Cloisters, St Luke’s Campus, Exeter, EX1 2LU UK

**Keywords:** School attendance, Absenteeism, Truancy, Emotional disorder, Anxiety, Depression

## Abstract

Emotional disorder may be associated with absence from school, but the existing evidence is methodologically weak. We studied the relationships between anxiety, depression and emotional difficulties, and school absence (total, authorised and unauthorised) using data from the 2004 British Child and Adolescent Mental Health Survey (BCAMHS). The BCAMHS was a cross-sectional, community survey of 7977 5- to 16-year-olds. Emotional disorder was assessed using the Development and Wellbeing Assessment (DAWBA), and emotional difficulties using the Strengths and Difficulties Questionnaire (SDQ) completed by teachers and parents. Teachers reported days absent in the previous school term. Multivariable negative binomial regression was used to examine the impact of emotional disorder and difficulties on absence. Age, gender and general health were explored as moderators. Anxiety, depression and emotional difficulties were associated with higher rates of all types of absence [rate ratios for total absence: anxiety 1.69 (1.39–2.06) *p* < 0.001; depression 3.40 (2.46–4.69) *p* < 0.001; parent-reported emotional difficulties 1.07 (1.05–1.10) *p* < 0.001; teacher-reported emotional difficulties 1.10 (1.08–1.13) *p* < 0.001]. The strongest association was observed for depression and unauthorised absence. Relationships were stronger for secondary compared to primary school children. Health and educational professionals should be aware that children with poor attendance may be experiencing emotional ill health, regardless of absence type. The absence may provide a useful tool to identify those who require additional mental health support. Findings highlight the widespread burden of emotional disorder and the need to support those with emotional ill health in continuing to access education.

**Electronic supplementary material:**

The online version of this article (10.1007/s00787-019-01342-4) contains supplementary material, which is available to authorized users.

## Introduction

Emotional disorders are among the most common psychiatric disorders in children and adolescents, with worldwide point prevalence estimates of 7% for anxiety and 3% for depression [1]. Both anxiety and depression are among the leading contributors to the burden of disease in children and adolescents worldwide [2]. In addition to causing substantial distress, childhood emotional disorders are associated with a range of adverse outcomes including educational failure, physical health problems, risk-taking behaviour, adult mental illness, substance abuse and increased risk of suicide [3–7]. Furthermore, onset of emotional disorder during childhood or adolescence is associated with greater functional impairment in a range of domains compared to adult-onset disorder [8]. Despite this, approximately 80% of children and adolescents with emotional disorders do not access services, a figure greater than that observed for other psychiatric disorders [9, 10].

The UK government’s recent Green Paper recognises the important role that schools have to play in identifying mental ill health at an early stage, supporting students who are experiencing difficulties, and referring to specialist support services where necessary [11]. However, a 2018 report by the UK Department for Education found that only 3 out of 90 (3%) schools surveyed had policies in place specifically regarding students’ mental health. Furthermore, those that did have policies in place used disruptive behaviour as their main way of identifying students with mental health needs [12], which is most likely to miss those with internalising problems such as depression or anxiety. Furthermore, universal screening approaches for the identification of emotional disorder in schools produce a high number of false positives and may lack efficiency [13]. Therefore, new ways are needed to identify children and adolescents with emotional ill health.

Previous studies have suggested that poor school attendance may be a sign of emotional disorder [14–16], and a recent systematic review concluded that anxiety and depression are associated with higher rates of school absence [17, 18]. However, that systematic review identified substantial weaknesses with the current evidence base, including poor methodological quality, a lack of comprehensive studies in UK populations, and few studies that have reported associations with authorised or excused absences, despite this being the most common type of absence. In addition, few studies have investigated the relationship for different subgroups of children such as those of a particular age, or for girls compared to boys, and there have been no formal moderator analyses that we are aware of. Age, in particular, should be investigated as a moderator, given that the prevalence of emotional disorder and the rate of school absence are greater in adolescents compared to younger children [19–21].

A complicating factor in the field of school attendance is the widespread lack of consensus regarding terminology and definitions. For example, “truancy” may refer to pupils who are absent due to a lack of interest in school or defiance of authority and who attempt to conceal the absence from their parents. Researchers and policy-makers, however, frequently use “truancy” to refer to unauthorised absences in general [22–24, 16]. In contrast to truancy, “school refusal” is commonly used to describe pupils who miss school due to anxiety or emotional distress and who do not typically attempt to conceal the absence from their parents. Truancy and school refusal are often considered to be associated with externalising and internalising disorders, respectively, although it is important to note that this may not always be the case [14].

However, research has shown that school refusal and truancy are not mutually exclusive [14], and some researchers call for use of broader terms that do not make assumptions about the underlying aetiology of the problem [25, 26]. In education policy and practice, absences are commonly separated into authorised and unauthorised absences [19], and the dataset used in the present study utilises this framework. However, it is important to note that authorised and unauthorised absences may also be subject to inconsistencies. For example, it is likely that the decision to authorise (or not) an absence will vary between schools and between individual staff members. Given that standardised definitions of authorised and unauthorised absence exist [27], we consider that such inconsistencies are likely to be less impactful than for other terms such as “truancy” and “school refusal”.

We undertook a secondary analysis of the 2004 British Child and Adolescent Mental Health Survey (BCAMHS) [21], which is a large, nationally-representative dataset that spans from 5 to 16 years. Although previous research has suggested that behavioural disorders are also related to school absence, particularly unauthorised absence or truancy [28, 29], the present study focuses on anxiety and depression because these disorders are so frequently unrecognised by adults around the child, particularly in education settings [30]. The BCAMHS benefits from having diagnostic measures of emotional disorder in addition to measures of emotional symptoms and school absence. We predicted that anxiety, depression and emotional difficulties would be associated with higher rates of total, authorised and unauthorised school absence. In addition, we explored gender, age and general health as moderators of these associations.

## Methods

The original BCAMHS surveys had approval from Medical Research Ethics Committees (MRECs), and ethical approval for this secondary analysis was granted by the University of Exeter Medical School Ethics Committee. Full details of the methods and sampling frame for the 2004 BCAMHS are available elsewhere [21], but a summary is provided here.

### Sample

**Fig. 1:**
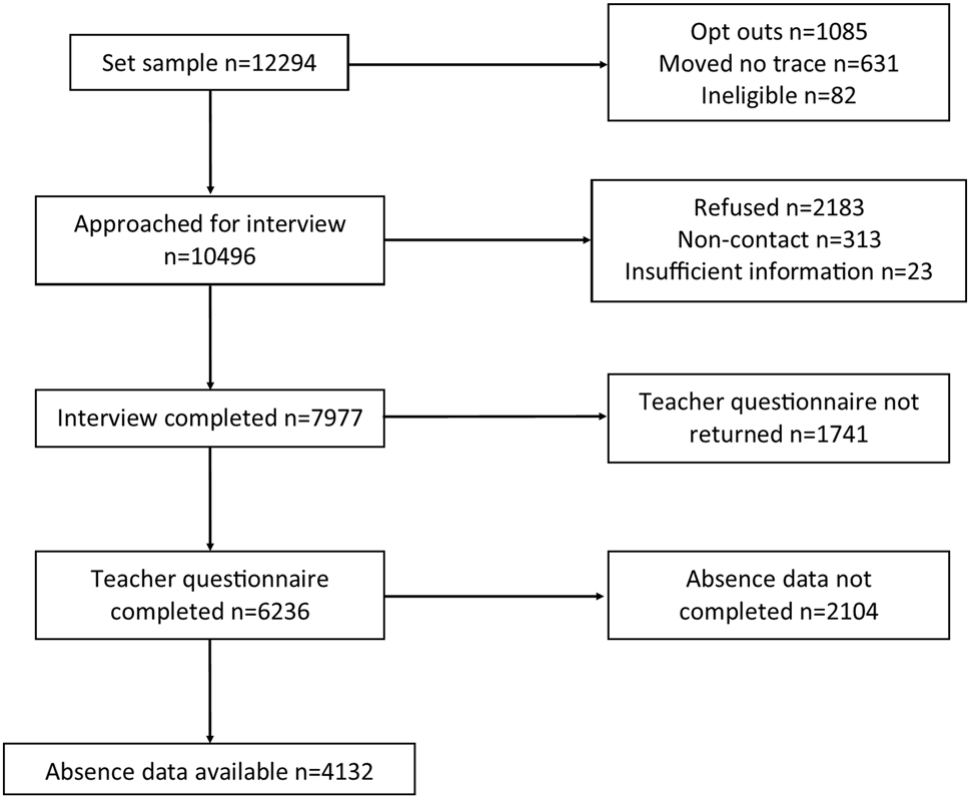
Flow diagram showing recruitment to the 2004 British Child and Adolescent Mental Health Survey

The BCAMHS involved a representative sample of children and young people aged 5–16 years, living in private households in Great Britain, sampled via the Child Benefit register. In 2004, Child Benefit was available to all British parents on a per-child basis, and had nearly complete take-up. Four hundred and twenty-six postal sectors were sampled by the Office for National Statistics, with a probability related to the size of the sector, and stratified by regional health authority and social economic group. A target sample of 12,294 children was selected and, after removing those addresses that opted out or were ineligible, 10,496 families were approached, and 7977 completed a baseline interview (see Fig. 1). The BCAMHS used a multi-informant model, with parents (*N* = 7977) and children aged 11 years and above (*N* = 3344) completing a face-to-face interview, and a postal questionnaire sent to teachers where parents gave consent (*N* = 6236).

## Measures

### Anxiety and depression

The Development and Wellbeing Assessment was used to assess the presence of psychiatric disorders according to criteria in Diagnostic and Statistical Manual of Mental Disorders Fourth Edition (DSM-IV) [31]. The DAWBA is a validated standardised diagnostic interview that combines structured and open-ended questions [20, 21, 32]. The structured questions relate to DSM-IV diagnostic criteria, and these are complemented with open-ended questions and supplementary prompts where problems are identified. In the 2004 BCAMHS, the DAWBA was completed by parents, children aged 11 or over, and if the family agreed, the child’s teacher. Computer-generated summaries and predictions of likely psychiatric diagnoses were reviewed by a small group of experienced clinical raters, who could accept or overturn the computer-generated diagnoses. Clinical raters worked independently, with regular group discussion of difficult or borderline cases. The aim of the DAWBA is to replicate the process of clinical diagnosis as closely as possible [20]. The κ statistic for chance-corrected agreement between two clinicians who independently rated 500 children was 0.86 for any disorder [standard error (SE) 0.04], 0.57 for internalising disorders (SE 0.11), and 0.98 for externalising disorders (SE 0.02) [20]. A validation study demonstrated excellent discrimination between community and clinical samples in rates of diagnosed disorder, and substantial agreement between DAWBA and case note diagnoses in the clinical sample [32]. For the purposes of the current study, we used diagnosis of any anxiety disorder and diagnosis of any depressive disorder according to DSM-IV criteria.

### Emotional difficulties

Emotional difficulties were measured in the BCAMHS using the Strengths and Difficulties Questionnaire, which is a validated questionnaire that screens for common childhood psychopathology (Cronbach alpha 0.73, test–retest reliability 0.62; [33]). The SDQ comprises 25 items in five subscales: emotional problems, conduct problems, hyperactivity, peer problems, and prosocial behaviour. In the 2004 BCAMHS, all parents, teachers and children over 11 years were invited to complete the SDQ. For the purposes of the current study, the emotional problem subscale, as reported by parents and teachers, was used. We did not include child-reported SDQ scores due to extensive missing data when combined with teacher-reported absence (see “Missing data” below).

The emotional problems score ranges from zero to ten, with a higher score reflecting greater difficulties. A four-band categorisation has been created, which classifies scores as “close to average” (approximately 80% of the population), “slightly raised” (10% of the population), “high” (5% of the population), and “very high” (5% of the population) [34]. For this study, we used emotional difficulties as a continuous measure for the main analyses, but to improve statistical power for moderator analyses, the four-level categorical variable was used.

### School absence

When the parent and, if appropriate, child interviews were completed, parents were asked for consent to contact the child’s teacher, and nominated the teacher they felt knew the child best. Questionnaires asked teachers to report, to the nearest half day: (1) “How many days was the child absent during the last whole term?” and (2) “Of these absences, how many were unauthorised absences?” No definition of “unauthorised absences” was provided, but this is generally considered to mean any absence for which the school is not satisfied with the reason given [27]. For the current study, authorised absences were calculated by subtracting unauthorised from total absence. Authorised absence means that the school has either given approval in advance, or has accepted an explanation offered afterwards as justification for the absence, and includes illness, medical appointments, religious occasions and other exceptional circumstances [27].

Of the 6236 teacher questionnaires completed, 4132 answered at least one of the two absence questions, and 4024 answered both. Nine teachers reported the number of absences to be far in excess of the maximum number of days in any school term. A search of the Department for Education website (https://www.gov.uk/school-term-holiday-dates) suggested that schools rarely exceed 70 days of teaching in the spring term, when the majority of BCAMHS data was collected. The maximum number of absences was, therefore, limited to 70, and observations greater than this (*n* = 9) were recorded as missing.

### Background and sociodemographic characteristics

Background information collected included the child’s age, gender, ethnicity, number of stressful life events (e.g. death of a friend or family member, parental marital separation), mother’s highest educational qualification, and family structure (traditional, single parent, reconstituted or other). Housing tenure was used as a measure of socioeconomic status and, in line with previous work [35], was categorised according to whether families owned or rented their home. Learning difficulty was assessed by asking parents and teachers to estimate the child’s mental age as a percentage of their chronological age. Children were deemed to have a severe, moderate, borderline, or no learning difficulty if their parent or teacher estimated their mental age to be 40% or less, 60% or less, 80% or less, or more than 80% of their chronological age, respectively [36]. Parental mental health was assessed using the 12-item General Health Questionnaire (GHQ; [37]), and parents were asked to rate their child’s general health on a five-point scale from very good to very bad.

### Analysis

Analysis was conducted using Stata/SE 14.2 [38]. Absence and background information for children with no psychiatric disorder, any anxiety disorder, and any depressive disorder were summarised using means and standard deviations for continuous variables, and numbers and percentages for categorical variables. These groups were not mutually exclusive, since some children had both an anxiety and depressive disorder.

### Main analyses

Negative binomial regression was used to investigate the associations between emotional disorder (assessed via the DAWBA) and emotional difficulties (assessed via the SDQ) as exposure variables, and total, authorised and unauthorised school absences as outcome variables. Negative binomial (rather than Poisson) regression, and robust standard errors, was used due to over-dispersion in the data [39]. Potential confounders were identified from previous literature and theory, and were tested in a single multivariable model with absence as the outcome. Those variables that were significant predictors at the 5% level were included as confounders in final multivariable models, and these were: age, gender, ethnicity, housing tenure, mother’s highest educational qualification, learning difficulty, stressful life events, and family type. We conducted a sensitivity analysis to determine the impact of including two additional variables that were not included in our primary analyses as we believed they might lie on the causal pathway between emotional disorder and school absence (parental mental health and child’s general health).

### Moderator analyses

After conducting our main analyses as described above, moderator analyses were conducted by including interaction terms in univariable and multivariable negative binomial regression models. Multivariable models included all confounders used in the main analyses. For each moderator, Wald tests were used to determine the statistical significance of the interaction term and, if statistically significant, the main analysis was repeated separately for each subgroup of the moderator.

The following variables were specified a priori as potential moderators:

- Gender
- School level (primary or secondary): used as a proxy for age. This was derived using the child’s age and month of birth, with children classed as “primary” if they were in school years reception to 6 (ages 5–11), and “secondary” if they were in school years 7–11 (ages 11–16).
- General health: given the lack of previous research in this regard, we believed it possible that general health could be either a moderator or mediator of the association between emotional disorder and school absence. We tested general health as a moderator, collapsed into a binary variable (*very good or good* versus *fair, bad or very bad*) due to no or few participants with anxiety or depression in some categories of the original five-level variable.

### Missing data

There was a substantial amount of missing data for our main outcome variables (48.2% missing for total, 49.6% for unauthorised, and 49.7% for authorised absence), and we, therefore, used multiple imputation on the assumption that data were missing at random (MAR) according to Rubin’s rules, i.e. that missingness was accounted for by other variables within the dataset [40]. Multiple imputation adjusts for the bias and loss of statistical power that occurs in analyses restricted to participants with complete data [41]. Missing data were imputed using the chained equation approach with Stata’s *mi impute chained* command. Predictive mean matching, in which imputed values are sampled only from the observed values, was used to impute absence and emotional difficulties scores, since these variables were not normally distributed [42]. Fifty imputed datasets were created as per good-practice guidelines to impute 100 times the fraction of missing information [42].

Variables used to impute missing data included all exposures, outcomes and confounders, as well as family functioning measured with the McMaster Family Activity Device [43], mother’s age when the child was born, teacher-reported age level of the child, household income, whether the child felt picked on by a teacher, whether the child had any physical disorder, and if a parent had experienced a serious physical or mental condition since the child’s birth. A sensitivity analysis was performed by repeating all analyses with complete cases only. Moderator analysis was performed using only complete case data, as it was not possible to include interaction terms in the imputation model due to there being no or very few cases with emotional disorder in some variable levels.

## Results

### Sample characteristics

Of the 7977 children in the sample, 7213 (90.4%) had no psychiatric disorder, 263 (3.3%) had an anxiety disorder, and 68 (0.9%) had a depressive disorder. These groups are not mutually exclusive since 38 children (0.5%) had both an anxiety and depressive disorder. The remaining 471 children (5.9%) had a disorder other than anxiety or depression and are not included in this analysis. Table 1 summarises the characteristics of children according to their disorder status. Children and adolescents with anxiety had a greater mean number of teacher-reported total, authorised and unauthorised absences than those with no disorder, and children and adolescents with depression had an even greater number of absences. Children for whom absence data were missing differed in several domains to those for whom absence data were available (see Supplementary Material), but bias was minimised by including all of these variables in multiple imputation models [41].

**Table 1:** Characteristics of children with no psychiatric disorder, any anxiety disorder, and any depressive disorder

	No disorder (*N* = 7213)	Any anxiety disorder (*N* = 263)	Any depressive disorder (*N* = 68)
School absence^a^: mean (SD)			
Total	3.8 (5.9)	8.1 (10.8)	17.5 (16.2)
Authorised	3.3 (5.1)	6.7 (9.1)	10.1 (11.1)
Unauthorised	0.44 (2.4)	1.5 (6.0)	7.4 (4.2)
Age in years: mean (SD)	10.5 (3.4)	11.6 (3.4)	13.4 (2.5)
Gender: *n* (%)			
Male	3641 (50.5)	118 (44.9)	25 (36.8)
Female	3572 (49.5)	145 (55.1)	43 (63.2)
Ethnicity: *n* (%)			
White	6232 (86.5)	232 (88.2)	60 (88.2)
Ethnic minority	977 (13.5)	31 (11.8)	8 (11.7)
Housing tenure: *n* (%)			
Own home	5268 (73.1)	130 (49.4)	35 (51.5)
Rented	1940 (26.9)	133 (50.6)	33 (48.5)
Mother’s highest qualification: *n* (%)			
Degree or diploma	1954 (27.8)	36 (14.2)	14 (21.2)
A-level or good GCSE	2969 (42.2)	91 (36.0)	18 (27.3)
Poor GCSE or other	932 (13.3)	43 (17.0)	11 (16.7)
None	1174 (16.6)	83 (32.8)	23 (34.8)
Learning difficulty: *n* (%)			
No	6677 (93.1)	196 (75.4)	52 (77.6)
Borderline, moderate or severe	493 (6.9)	64 (24.6)	15 (22.4)
Stressful life events: mean (SD)	0.9 (1.1)	2.0 (1.5)	2.3 (1.1)
Family structure: *n* (%)			
Traditional	4770 (66.1)	111 (42.2)	26 (38.2)
Single parent, reconstituted, or other	2443 (33.9)	152 (57.8)	42 (61.8)
Child’s general health: *n* (%)			
Very good or good	6762 (93.7)	212 (80.6)	45 (66.2)
Fair, bad or very bad	344 (4.8)	49 (18.6)	22 (32.4)
Parental mental health^b^: mean (SD)	1.4 (2.5)	4.0 (3.9)	5.1 (4.2)

Based on 7977 initial sample; 7213 children had no psychiatric disorder, 263 had an anxiety disorder and 68 had a depressive disorder. Thirty-eight children had both anxiety and depression; hence, these two columns are not mutually exclusive

^a^Absence refers to the number of days absent in the previous whole school term, as reported by teachers

^b^Parental mental health was assessed using the General Health Questionnaire, a screening questionnaire for psychiatric disorder in the general population; higher scores reflect more symptoms

### Main analyses

Table 2 provides results of regression models comparing the rate of teacher-reported absence between disorder and no disorder groups, as well as the rate of absence for each one-point increase on the SDQ emotional difficulties scale. Sensitivity analysis using only cases with complete data demonstrated similar effect estimates to those produced with multiply imputed data, but the latter resulted in more precise estimates (i.e. narrower confidence intervals). Therefore, results presented here are those obtained from analysing imputed data, but results from complete case analysis are available in supplementary material. Sensitivity analysis was also performed to test the impact of including parental mental health and child’s general health as confounders. Including these variables resulted in minor reductions in effect estimates but did not change the overall conclusions. Results presented here are from analyses that were not adjusted for parental mental health or child’s general health.

**Table 2:** Rate of school absence according to emotional disorder status and parent- and teacher-reported emotional difficulties scores

	Total absence		Authorised absence		Unauthorised absence	
	Rate ratio and 95% CI	*p* value	Rate ratio and 95% CI	*p* value	Rate ratio and 95% CI	*p* value
Anxiety disorder						
Unadjusted	2.21 (1.82–2.67)	<0.001	2.03 (1.67–2.47)	<0.001	3.52 (1.94–6.39)	<0.001
Adjusted	1.69 (1.39–2.06)	<0.001	1.61 (1.32–1.97)	<0.001	2.23 (1.19–4.15)	0.012
Depressive disorder						
Unadjusted	4.59 (3.41–6.17)	<0.001	3.13 (2.18–4.51)	<0.001	16.55 (9.03–30.32)	<0.001
Adjusted	3.40 (2.46–4.69)	<0.001	2.39 (1.63–3.50)	<0.001	11.24 (5.40–23.39)	<0.001
Parent-reported emotional difficulties						
Unadjusted	1.11 (1.08–1.13)	<0.001	1.10 (1.08–1.12)	<0.001	1.14 (1.07–1.21)	<0.001
Adjusted	1.07 (1.05–1.10)	<0.001	1.07 (1.05–1.09)	<0.001	1.08 (1.00–1.15)	0.048
Teacher-reported emotional difficulties						
Unadjusted	1.13 (1.10–1.15)	<0.001	1.12 (1.09–1.14)	<0.001	1.20 (1.12–1.28)	<0.001
Adjusted	1.10 (1.08–1.13)	<0.001	1.09 (1.07–1.12)	0.008	1.13 (1.06–1.22)	0.001

Based on 7977 initial sample; 7213 children had no psychiatric disorder, 263 had an anxiety disorder and 68 had a depressive disorder. Anxiety and depression are binary predictors; emotional difficulties are continuous scores ranging from 0 to 10 and hence, the rate ratios represent the increase in rate of absence per one-point increase on the emotional difficulties scale. Adjusted estimates are adjusted for age, gender, ethnicity, housing tenure, mother’s highest educational qualification, learning difficulty, stressful life events, and family type

*CI* confidence interval

### Anxiety and depression as predictors of school absence

Children with any anxiety disorder had a higher rate of total [adjusted incident rate ratio (IRR) 1.69, 95% CI 1.39–2.06, *p* < 0.001], authorised (adjusted IRR 1.61, 95% CI 1.32–1.97, *p* < 0.001) and unauthorised (adjusted IRR 2.23, 95% CI 1.19–4.15, *p* = 0.01) teacher-reported absences compared to children with no disorder. The association for depression was even greater, with the rate of total (adjusted IRR 3.40, 95% CI 2.46–4.69, *p* < 0.001), authorised (adjusted IRR 2.39, 95% CI 1.63–3.50, *p* < 0.001), and unauthorised (adjusted IRR 11.2, 95% CI 5.4–23.4, *p* < 0.001) absences higher than for those with no disorder.

### Parent-reported emotional difficulties as a predictor of school absence

Higher scores on the parent-reported emotional difficulties subscale of the SDQ were associated with a higher rate of all three types of absence. These relationships remained statistically significant after adjusting for confounders (IRR for total absence 1.07, 95% CI 1.05–1.10, *p* < 0.001; authorised absence 1.07, 95% CI 1.05–1.09, *p* < 0.001; unauthorised absence 1.08, 95% CI 1.00–1.15, *p* = 0.048). These rate ratios refer to the increase in the rate of teacher-reported absence per one-point increase on the parent-reported emotional difficulties scale.

### Teacher-reported emotional difficulties as a predictor of school absence

Higher scores on the teacher-reported emotional difficulties subscale of the SDQ were also associated with higher rates of all three types of absence, both in unadjusted and adjusted analyses (adjusted IRR for total absence 1.10, 95% CI 1.08–1.13, *p* < 0.001; authorised absence 1.09, 95% CI 1.07–1.12, *p* = 0.008; unauthorised absence 1.13, 95% CI 1.06–1.22, *p* = 0.001). These rate ratios refer to the increase in the rate of absence per one-point increase on the teacher-reported emotional difficulties scale.

### Moderator analyses

Results from all tests of interaction are provided in supplementary material, and a summary of pertinent findings is presented here.

### Gender

There was no evidence that gender moderated the relationship between any of our predictors and outcomes (all *p* values > 0.1).

### School level

**Fig. 2:**
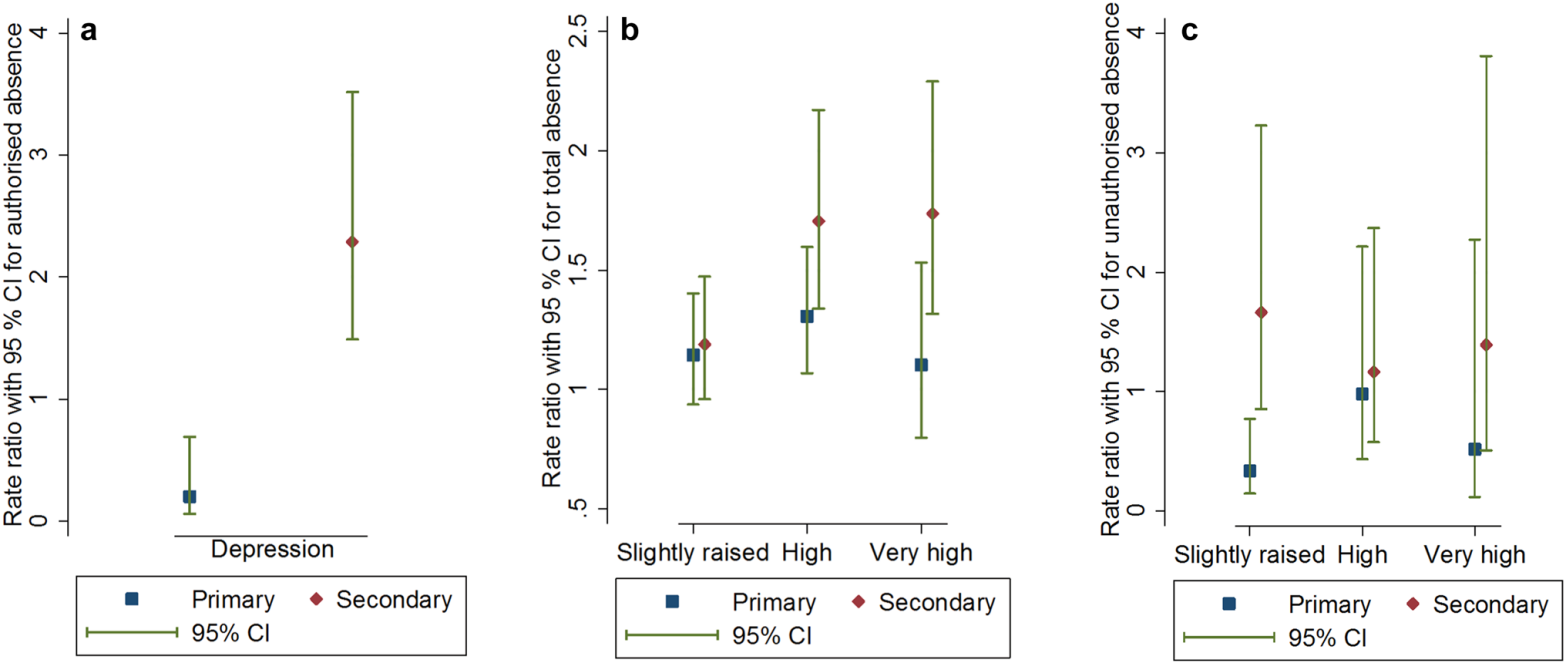
School level status (primary versus secondary) as a moderator of the associations between: **a** depression and authorised absence (graph displays rate ratio for authorised absence comparing children with depression to those with no psychiatric disorder). **b** Parent-reported emotional difficulties and total absence (graph displays rate ratios for total absence comparing children with slightly raised, high and very high emotional difficulties scores to those with close to average scores). **c** Parent-reported emotional difficulties and unauthorised absence (graph displays rate ratios for unauthorised absence comparing children with slightly raised, high and very high emotional difficulties scores to those with close to average scores)
- Parent-reported emotional difficulties and total absence (adjusted interaction test *p* = 0.04). Subgroup analysis again demonstrated a stronger relationship for secondary than for primary school children, particularly for children whose parents scored them “very high” (adjusted IRR primary: 1.10, 95% CI 0.80–1.53; secondary: 1.74, 95% CI 1.32–2.29) or “high” (adjusted IRR primary: 1.31, 95% CI 1.07–1.60; secondary: 1.70, 95% CI 1.34–2.17) on the emotional difficulties scale (see Fig. 2).
- Parent-reported emotional difficulties and unauthorised absence (adjusted interaction test *p* = 0.003). Subgroup analysis demonstrated a stronger relationship for secondary compared to primary school children, although in this case the difference between school levels was most pronounced for children whose emotional difficulties scores were “slightly raised” (primary: adjusted IRR 0.34, 95% CI 0.15–0.77; secondary: adjusted IRR 1.66, 95% CI 0.86–3.23) (see Fig. 2).

School level, used as a proxy for age, was a statistically significant moderator of the following associations:

- Depression and authorised absence (adjusted interaction test *p* < 0.001). Subgroup analysis demonstrated a stronger relationship for secondary (adjusted IRR 2.29, 95% CI 1.49–3.52) than for primary (adjusted IRR 0.20, 95% CI 0.06–0.69) school children (see Fig. 2).

Overall, these moderator analyses suggest that the association between emotional disorder and school absence may be greater for secondary compared to primary school students.

### General health

**Fig. 3:**
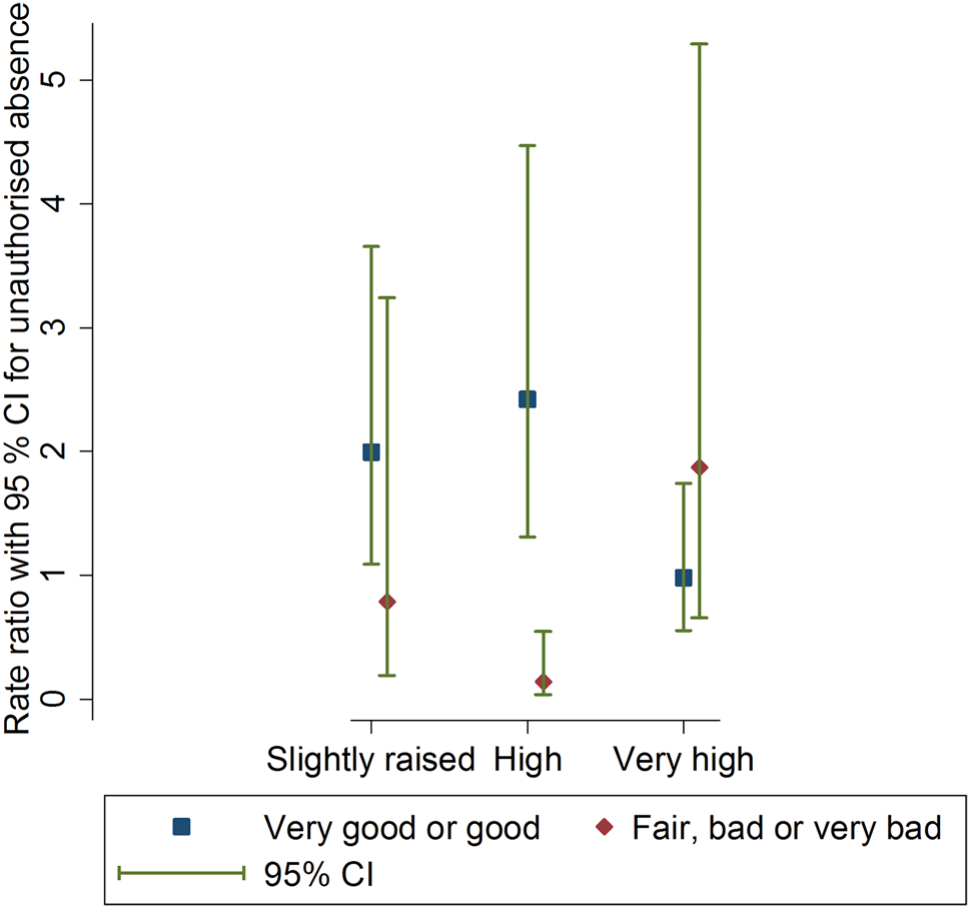
Graph to show general health (*very good or good* versus *fair, bad or very bad*) as a moderator of the association between teacher-reported emotional difficulties and unauthorised absence (graph displays rate ratios for children with slightly raised, high or very high, compared to close to average emotional difficulties scores)

General health was a statistically significant moderator of the relationship between teacher-reported emotional difficulties and unauthorised absence (adjusted interaction test *p* < 0.001). Subgroup analysis demonstrated that the association was greater for children with good health compared to those with bad health. The difference in subgroups was particularly pronounced for children whose teacher scored them “high” on the emotional difficulties subscale (good health: adjusted IRR 2.42, 95% CI 1.31–4.47; bad health: adjusted IRR 0.14, 95% CI 0.04–0.55) (see Fig. 3).

## Discussion

We found evidence for associations between anxiety, depression and emotional difficulties with total, authorised and unauthorised absences in UK children and adolescents aged 5–16. All four measures of emotional disorder/difficulties were associated with an increased risk of all three types of school absence. These findings were in line with our expectations and previous evidence [14–18].

This was the first study to demonstrate consistent relationships across all types of school absence (total, authorised and unauthorised) and several measures of emotional disorder. That said, the associations were greater for unauthorised compared to authorised absences, particularly in relation to depression, where children and adolescents with depression had eleven times the rate of unauthorised absence in the previous school term compared to their peers with no psychiatric disorder. The extent of this relationship is surprising given the long-held belief that unauthorised absence is associated with behavioural disorders rather than anxiety or depression [44, 45]. Although 18 (26.5%) of the 68 children with depression in this sample also had a conduct or oppositional disorder, the majority of them did not, and thus it is unlikely that the association between depression and unauthorised absence is simply a result of comorbid behavioural disorders. These findings are also in line with a recent systematic review that reported particularly strong evidence with regard to depression and unexcused absence or truancy [17].

It is interesting that associations with all three measures of absence were greater for depression compared to anxiety, a finding that replicates those from previous research [14, 15, 46, 47]. It is possible that symptoms of depression such as difficulties with concentration and lack of motivation lead to greater impairments in education compared to symptoms of anxiety. A previous study demonstrated that the majority of young people (78%) with high symptoms of anxiety do not meet Kearney’s criteria for problematic absenteeism (i.e. miss at least 25% of school time for at least 2 weeks, experience difficulty attending classes for at least 2 weeks with significant interference with the child’s routine, and are absent for at least 10 days during any 15-week period) [48]. It may be that young people with anxiety, compared to those with depression, are more able to continue attending regularly despite their symptoms. However, it is important to note that the present study did not compare rates of absence for those with depression versus anxiety, and future research designed to make this direct comparison would help to further our understanding in this respect.

Findings suggest that parents, clinicians and school staff should be aware that high rates of school absence, whether authorised or unauthorised, may be a sign of underlying emotional ill health, requiring assessment and, if necessary, intervention or referral to more specialist services. Importantly, health and education professionals should not assume that unauthorised absence is necessarily a signifier of behavioural difficulties, but may also indicate that a young person is experiencing anxiety and/or depression.

Given the UK government’s recent proposals for schools to play a greater role in supporting students’ mental health [11], our findings suggest that school attendance could serve as a simple and easy method for identifying students who may be experiencing emotional ill health. However, there are no studies that we are aware of that have explicitly investigated the effectiveness of attendance data to identify emotional disorders in school settings. Given that universal screening approaches produce a high number of false positives [13], this is an important topic for future research.

Both parent- and teacher-reported SDQ emotional difficulties scores were associated with school absence. The SDQ may be used by schools as a universal screening tool for the identification of students with mental health difficulties [12, 49], but our findings also support a more targeted approach in which it is used to screen students with poor attendance, to identify those who may be experiencing mental health difficulties. This is especially important with respect to emotional disorder, given the low rates of treatment utilisation [9, 10] and that schools commonly use disruptive behaviour as their primary way of identifying students with mental health problems [12], which is likely to lead to under-recognition of anxiety and depression. Our findings also highlight the burden of childhood emotional disorder beyond healthcare settings, having the potential to adversely impact educational outcomes. Frequent absence from school is itself associated with a range of adverse consequences including poor academic outcomes, social isolation, economic deprivation and future unemployment [50–52], and thus it is crucial that steps are taken to support children and adolescents experiencing emotional ill health to continue to access education.

Our moderator analyses provided no evidence that these associations are different for boys and girls. There was, however, evidence that the association may differ according to age. Specifically, the associations between (a) depression and authorised absence, (b) parent-reported emotional difficulties and total absence, and (c) parent-reported emotional difficulties and unauthorised absence were greater for secondary than for primary school students. There is evidence that somatic symptoms related to emotional disorders are more common with increasing age [53, 54], and it may be that somatic symptoms result in greater school absence for adolescents compared to younger children with these disorders. However, general health was only a statistically significant moderator for teacher-reported emotional difficulties predicting unauthorised absence, and not for any other of our measures of emotional disorder or absence. It, therefore, seems unlikely that the moderator effect observed for age is driven by differences in somatic symptoms. It is, however, possible that emotional disorder has less of an impact on school attendance for younger children because their attendance is largely determined by parents/carers, whereas adolescents may have greater ownership over their own attendance.

It is unclear why the association between teacher-reported emotional difficulties and unauthorised absence would be greater for children with good compared to bad health. It is possible that for children whose general health is poor, their difficulty attending school may be attributed by those around them to their general health, and such absences may be more likely to be authorised. However, general health was not found to moderate the association between any of our emotional disorder measures and authorised absences, so this seems unlikely.

### Strengths and limitations

This was the first study to comprehensively investigate the association between emotional disorder and school absence in a UK population of children and adolescents, and we addressed many of the limitations of previous research. The BCAMHS benefits from a large, nationally representative sample spanning the entire age range of compulsory education in the UK, and the use of clinical diagnoses in addition to multi-informant symptom questionnaires is a strength. A population survey such as this has the additional strength that it is likely to have included children with the full spectrum of school attendance, as opposed to studies that have relied on school-based data collection, which is likely to exclude those with the poorest attendance. This was the first study that we are aware of to formally investigate gender, age and general health as moderators of the association between emotional disorder and school absence, enabling us to report on the effects for subgroups of the population. Our models adjusted for several factors known to be associated with school absence, minimising the likelihood that the effects were due to confounding.

Despite the large initial sample of the BCAMHS, absence was teacher reported and thus there were substantial missing data for our main outcome measures, and exploration of missing data established that missingness was not completely at random. However, we used multiple imputation to overcome the bias inherent with such missingness [41]. We were unable to use multiple imputation for our moderator analyses because the introduction of interaction terms to the imputation model affected its stability, due to small case numbers in individual levels of several variables. However, given that sensitivity analysis for our main effects demonstrated that multiple imputation improved the precision of effect estimates but did not substantially change the estimates, we consider it unlikely that performing the moderator analyses with imputed data would have resulted in alternative conclusions.

We reported findings separately for total, authorised and unauthorised absences, allowing us to draw conclusions in relation to subtypes of absence as well as absence overall. The use of teacher-reported absence data could be considered a strength in comparison with previous research which has tended to use child reports, which may be less reliable. However, it is unclear to what extent teachers in the BCAMHS used administrative data rather than relying on recall to complete absence information. The lack of definition provided to teachers regarding unauthorised absence is also a limitation, and it is possible that teachers were unaware of the standard definition for unauthorised absence utilised by the Department for Education, and that the decision to record an absence as unauthorised may differ between schools and between individual teachers. Furthermore, because teachers did not report the total number of available days, we selected a maximum number (*N* = 70) that we considered reasonable for any school term; however, for some individuals this will have been an over- or under-estimate. It is likely that all methods of measuring school absence introduce some degree of bias, and future research should ideally utilise multiple methods to reduce the impact of measurement error.

A final important limitation of the current study is the cross-sectional nature of the data. Thus, we were only been able to demonstrate associations between emotional disorder and school absence, and cannot draw any conclusions about the direction of the relationships, nor can we make any claims regarding causality. There have been few longitudinal studies to explore this relationship [55, 56], and none that we are aware of that have explored anxiety and depression as predictors of subsequent absence, as well absence as a predictor of subsequent anxiety and depression. Future research utilising longitudinal data would help to establish whether absence or emotional ill health comes first.

## Conclusions

We found evidence of associations between several measures of emotional disorder and absence from school. Clinical and educational professionals should be aware that a child with poor attendance may be experiencing underlying emotional ill health, whether or not those absences are authorised or unauthorised. School absence may be a useful tool to identify children and adolescents who are experiencing emotional difficulties; a group who are commonly under-recognised. Furthermore, our findings highlight the widespread burden of emotional disorder and the need to support children and adolescents with emotional ill health to continue to access education.

## Electronic supplementary material

Below is the link to the electronic supplementary material.

Supplementary file1 (DOCX 18 kb)
