# Supplementary material for: Emotional disorder and absence from school: findings from the 2004 British Child and Adolescent Mental Health Survey
Source: Eur Child Adolesc Psychiatry. 2019 May 3;29(2):187–98. doi: 10.1007/s00787-019-01342-4 (PMC7024694; doi:10.1007/s00787-019-01342-4)
Supplement: Supplementary file 1 — Supplementary file1 (DOCX 18 kb) [file 787_2019_1342_MOESM1_ESM.docx]

## Emotional disorder and difficulties and attendance at school: Findings from the 2004 British Child and Adolescent Mental Health Survey

Journal: European Child and Adolescent Psychiatry

Authors: Katie Finning, Tamsin Ford, Darren A Moore and Obioha C Ukoumunne

# Supplementary material

**Characteristics of participants for whom absence data was available compared to those for whom absence data was missing**

|  | **Absence data available (N=4123)** | | **Absence data missing (N=3854)** | |
| --- | --- | --- | --- | --- |
| **Age in years: Mean (SD)** | 10.27 (3.38) | | 10.83 (3.40) | |
| **Gender: N (%)** |  | |  | |
| Male | 2132 (51.6) | | 1979 (51.5) | |
| Female | 2000 (48.4) | | 1866 (48.5) | |
| **Ethnicity: N (%)** |  | |  | |
| White | 3616 (87.6) | | 3304 (86.0) | |
| Ethnic minority | 514 (12.5) | | 539 (14.0) | |
| **Housing tenure: N (%)** |  | |  | |
| Own home | 3010 (72.9) | | 2657 (69.2) | |
| Rented | 1120 (27.1) | | 1185 (30.8) | |
| **Mother’s highest qualification: N (%)** | |  | |  |
| Degree or diploma | 1153 (28.8) | | 913 (24.5) | |
| A-level or good GCSE | 1682 (41.6) | | 1534 (41.2) | |
| Poor GCSE or other | 517 (12.8) | | 544 (14.6) | |
| None | 678 (16.8) | | 734 (19.7) | |
| **Learning difficulty: N (%)** | |  | |  |
| No | 3738 (90.5) | | 3475 (91.5) | |
| Borderline, moderate or severe | 391 (9.5) | | 325 (8.6) | |
| **Stressful life events: Mean (SD)** | 0.97 (1.13) | | 1.08 (1.19) | |
| **Family structure: N (%)** |  | |  | |
| Traditional | 2742 (66.4) | | 2370 (61.6) | |
| Single-parent, reconstituted, or other | 1390 (33.6) | | 1475 (38.4) | |
| **Child’s general health: N (%)** |  | |  | |
| Very good or good | 3872 (94.9) | | 3529 (93.3) | |
| Fair, bad or very bad | 209 (5.1) | | 255 (6.7) | |
| **Parental mental health*: Mean (SD)** | 1.58 (2.61) | | 1.67 (2.69) | |

**Rate of school absence according to emotional disorder status and parent- and teacher-reported emotional difficulties scores, based on complete case data**

|  | **TOTAL ABSENCE** | | **AUTHORISED ABSENCE** | | **UNAUTHORISED ABSENCE** | |
| --- | --- | --- | --- | --- | --- | --- |
|  | **Rate ratio & 95% CI** | **p-value** | **Rate ratio & 95% CI** | **p-value** | **Rate ratio & 95% CI** | **p-value** |
| **Anxiety disorder** | | | | | | |
| Unadjusted | **2.15 (1.71 to 2.71)** | **p<0.001** | **2.01 (1.59 to 2.54)** | **p<0.001** | **3.30 (1.61 to 6.76)** | **p=0.001** |
| Adjusted | **1.61 (1.28 to 2.03)** | **p<0.001** | **1.55 (1.22 to 1.98)** | **p<0.001** | 1.51 (0.76 to 3.00) | p=0.235 |
| **Depressive disorder** | | | | | | |
| Unadjusted | **4.66 (3.28 to 6.62)** | **p<0.001** | **3.03 (2.00 to 4.60)** | **p<0.001** | **16.78 (7.78 to 36.18)** | **p<0.001** |
| Adjusted | **3.18 (2.20 to 4.59)** | **p<0.001** | **2.19 (1.38 to 3.45)** | **p=0.001** | **6.89 (2.80 to 16.92)** | **p<0.001** |
| **Parent-reported emotional difficulties** | | | | | | |
| Unadjusted | **1.11 (1.08 to 1.14)** | **p<0.001** | **1.12 (1.10 to 1.15)** | **p<0.001** | **1.24 (1.15 to 1.33)** | **p=0.001** |
| Adjusted | **1.07 (1.04 to 1.09)** | **p<0.001** | **1.07 (1.05 to 1.10)** | **p<0.001** | 1.01 (0.93 to 1.09) | p=0.854 |
| **Teacher-reported emotional difficulties** | | | | | | |
| Unadjusted | **1.14 (1.11 to 1.16)** | **p<0.001** | **1.12 (1.10 to 1.15)** | **p<0.001** | **1.24 (1.15 to 1.33)** | **p<0.001** |
| Adjusted | **1.10 (1.08 to 1.13)** | **p<0.001** | **1.10 (1.08 to 1.12)** | **p<0.001** | **1.11 (1.04 to 1.20)** | **p=0.003** |

**Results of Wald tests of interaction**

|  | **Total absences** | **Unauthorised absences** | **Authorised absences** |
| --- | --- | --- | --- |
| **GENDER** | | | |
| **Any anxiety disorder** | p=0.25  p=0.31 | p=0.14  p=0.58 | p=0.51  p=0.49 |
| **Any depressive disorder** | p=0.67  p=0.14 | p=0.49  p=0.38 | p=0.86  p=0.40 |
| **Emotional difficulties (parent-report)** | p=0.62  p=0.95 | p=0.16  p=0.89 | p=0.75  p=0.93 |
| **Emotional difficulties (teacher-report)** | p=0.77  p=0.54 | p=0.70  p=0.62 | p=0.21  p=0.15 |
| **SCHOOL LEVEL (*primary* versus *secondary*)** | | | |
| **Any anxiety disorder** | p=0.08  p=0.18 | p=0.56  p=0.39 | p=0.11  p=0.24 |
| **Any depressive disorder** | p=0.83  p=0.50 | p=0.07  p=0.15 | **p=0.002**  **p<0.001** |
| **Emotional difficulties (parent-report)** | **p=0.02**  **p=0.04** | **p<0.001**  **p=0.003** | p=0.10  p=0.08 |
| **Emotional difficulties (teacher-report)** | p=0.07  p=0.30 | p=0.13  p=0.53 | p=0.66  p=0.80 |
| **GENERAL HEALTH (*very good or good* versus *fair, bad or very bad*)** | | | |
| **Any anxiety disorder** | p=0.96  p=0.47 | p=0.19  p=0.43 | p=0.40  p=0.19 |
| **Any depressive disorder** | p-0.44  p=0.63 | p=0.92  p=0.97 | p=0.39  p=0.61 |
| **Emotional difficulties (parent-report)** | p=0.07  p=0.18 | p=0.09  p=0.09 | p=0.08  p=0.16 |
| **Emotional difficulties (teacher-report)** | p=0.62  p=0.71 | **p=0.01**  **p<0.001** | p=0.96  p=0.90 |
